# Is Recycled Polypropylene Suitable for Flame-Retarded Applications?

**DOI:** 10.1021/acsapm.6c00885

**Published:** 2026-06-12

**Authors:** Giulia Bernagozzi, Rossella Arrigo, Yue Xu, Miaojun Xu, Alberto Frache

**Affiliations:** 1 Department of Applied Science and Technology, 19032 Politecnico di Torino, Viale Teresa Michel 5, 15121 Alessandria, Italy; 2 National Interuniversity Consortium of Materials Science and Technology (INSTM), Via G. Giusti 9, 50121 Firenze, Italy; 3 Heilongjiang Key Laboratory of Molecular Design and Preparation of Flame Retarded Materials, College of Chemistry, Chemical Engineering and Resource Utilization, 47820 Northeast Forestry University, No. 26, Hexing Road, Xiangfang District, 150040 Harbin, China

**Keywords:** polypropylene, flame retardant, recycling, cone calorimeter, intumescent system

## Abstract

In this study, the performance of an intumescent flame-retardant (IFR) system in recycled polypropylene (PP) was investigated. To this aim, virgin and reprocessed PPs were melt-compounded with 21 wt % of an IFR consisting of piperazine pyrophosphate (PAPP) and melamine polyphosphate (MPP) (2:1 ratio), and the obtained materials were characterized in terms of morphology and rheological and combustion behavior. Cone calorimetry results showed that although both IFR-containing materials exhibited a similar reduction in heat release rate as compared to the unfilled matrices, the recycled PP-based system displayed a higher time to ignition and a pronounced delay in the second heat release rate peak. The observed behavior was explained by considering the formation of a more compact and denser char, likely promoted by the finer dispersion of IFR particles achieved in the low-viscosity recycled PP matrix. Furthermore, both materials achieved a V-0 rating in UL-94 testing and comparable LOI values. Finally, the assessment of the mechanical behavior of both systems demonstrated that the utilization of recycled PP does not compromise the tensile and flexural strength of the material, notwithstanding a slight decrease in ductility as compared to the virgin PP-based counterpart. Overall, these findings demonstrate that recycled PP can be effectively used in flame-retardant formulations, broadening its potential applications while reducing dependence on virgin materials and supporting the development of circular economy approaches.

## Graphical abstract

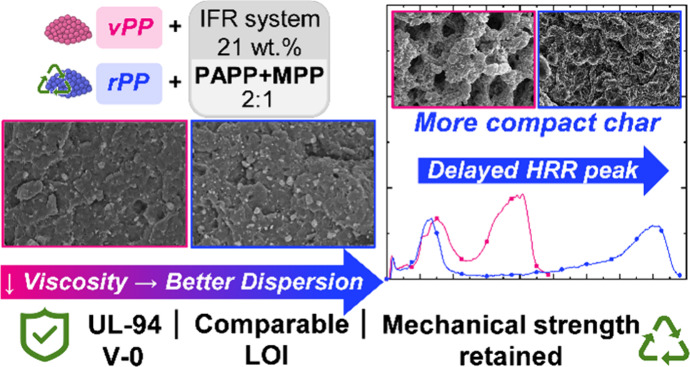

## 1 Introduction

When dealing with applications where plastics could be subjected to combustion and fire, the utilization of flame-retardant (FR) additives becomes a mandatory requirement. This is essential in order to delay the flashover time and increase the fire resistance of the materials.^1^ As a result, over the years, several commercial FRs have been developed to meet these demands. These additives can be differentiated based on their modes of action and chemical compositions,^2,3^ and their selection depends on various factors, including regulatory requirements, specific performance standards, and the potential fire scenarios that the materials are expected to face.

As highlighted in some recent reviews,^4^ current interest in the literature lies in investigating how plastics derived from recycling processes can be transformed into new polymeric formulations containing flame retardants, with the aim of upcycling plastics waste and producing enhanced functionality recycled products. In fact, the different forms of degradation undergone by polymers during their service life, as well as the thermomechanical degradation typically experienced during mechanical recycling processes, usually result in chemical and/or structural modifications of the macromolecular chains, thereby potentially impacting the FR effectiveness. Therefore, understanding the relationship between the degradation extent of a recycled polymer and its flame-retardant properties is a key factor for assessing the viability of using recyclates in fire-risk applications.

A few works dealing with the introduction of different FR systems in recycled polymers are reported in the literature.^5–9^ For instance, Casetta et al.^5^ investigated the flame retardancy of virgin and recycled PP/ethylene propylene rubber blends containing different contents of an intumescent flame retardant (IFR) system. The obtained results indicated an increase of the time to ignition and a decrease of the peak of heat release rate for recycled PP-based blends as compared to their virgin counterpart. Similarly, Bodzay et al.^6^ demonstrated the effectiveness of an IFR system on a PP-based waste derived from the light fraction of end-of-life vehicles, by achieving a decrease of the peak of heat release rate of about 80% as compared to recycled PP matrix. Also recycled PP-based composites containing wood flour, halogen-free flame retardants and synergistic agents with superior flame retardancy properties and mechanical performances were successfully developed by Ren et al.^7^

This work aims at evaluating the effectiveness of an intumescent flame-retardant system based on a mixture of piperazine pyrophosphate (PAPP) and melamine polyphosphate (MPP), in recycled PP (rPP). Although this IFR system has already been proven effective in enhancing the flame retardancy of PP-based materials,^10–15^ its performance in reprocessed matrices has not yet been systematically investigated. In particular, this study focuses on understanding how the microstructural modifications induced by repeated extrusion affect the rheological behavior, flame-retardant response, combustion behavior, and overall performance of polypropylene containing the IFR formulation. To this end, virgin PP (vPP) was subjected to 8 subsequent extrusion cycles in order to obtain a recycled PP with controlled composition and degradation extent, representative of the changes typically occurring during a mechanical recycling process. Subsequently, flame retarded vPP- and rPP-based materials were prepared by melt compounding both polymers with the IFR. After the preliminary microstructural and thermal characterization of the two materials, the fire retardancy performance of the materials was assessed by means of flammability tests (UL-94 and LOI), while their combustion behavior was investigated through cone calorimeter tests. The results revealed that the IFR system effectively enhanced flame-retardant performances in both virgin and recycled PP. Remarkably, while flammability remained practically unchanged, the rPP-based materials exhibited a different combustion behavior compared to their virgin counterparts, allowing establishing fundamental microstructure/flame retardancy relationships. Finally, in order to provide a comprehensive evaluation of the vPP- and rPP-based materials, their mechanical properties were also assessed.

## 2 Materials and Methods

### 2.1 Materials

Polypropylene MOPLEN HP500N from LyondellBasell, with a melt flow index of 12 g/10 min (230 °C/2,16 kg), was used as matrix (vPP). Recycled PP (rPP) was obtained by subjecting vPP to 8 extrusion cycles in a twin-screw extruder Leistritz ZSE 18 HP, selecting the following processing conditions: flat temperature profile at 210 °C, screw speed of 200 rpm and feed rate of 3 kg/h. The intumescent flame retardant (IFR) was prepared by mixing piperazine pyrophosphate (PAPP), supplied by Zhongshan Complord New Materials Co., Ltd., and melamine polyphosphate (MPP), obtained from Zhenjiang Senhua Flame Retardant Engineering Technology Co., Ltd., in a mass ratio of 2:1 (PAPP:MPP).

### 2.2 Processing

IFR-containing materials were obtained through melt processing in a twin-screw extruder (Leistritz ZSE 18 HP) at 900 rpm, with flow rate equal to 3 kg/h and the following temperature profile (°C): 160, 170, 180, 180, 180, 180. All the materials have been dried in an oven at 80 °C for 2 h before the processing. The IFR was introduced at 21 wt %. in both vPP and rPP.

The specimens for the further characterizations were prepared by injection molding, using a Haitian HTF86X 1 machine, with a temperature profile in the barrel equal to 165–175–180–180 °C and mold temperature of 30 °C.

### 2.3 Characterization Methods

The rheological behavior of all investigated materials was evaluated using a strain-controlled ARES rheometer (TA Instruments) equipped with parallel plate geometry (25 mm diameter). The tests were performed in nitrogen atmosphere to avoid the thermo-oxidative degradation of the sample during the measurement. Frequency sweep tests were performed at 180 °C at a frequency ranging from 100 to 0.1 rad/s within the linear viscoelastic region of the material (preliminarily assessed through strain sweep tests). The rheological tests were performed in triplicate.

The thermo-oxidative stability of all the samples was assessed using a thermogravimetric analyzer (TGA 8000, PerkinElmer) at a heating rate of 10 °C/min under air atmosphere. *T*_onset_ (temperature at which 2% of weight loss occurs), *T*_max_ (temperature at which maximum weight loss rate is observed in dTG-derivative-curves), and the residue at 600 °C were evaluated. The TGA tests were performed in triplicate and the results averaged.

Flame-retardant performances were evaluated by several standard methods. The limiting oxygen index (LOI) was determined with a JF-3 oxygen index tester (Jiangning, China) following ASTM D2863 standard and using specimens measuring 80 × 10 × 3 mm^3^. Vertical burning behavior (UL-94) was assessed with a CZF-5 horizontal and vertical combustion tester (China Jiangning Analytical Instrument Co.) in accordance with ASTM D3801, employing samples with dimensions equal to 130 × 13 × 3.2 mm^3^. The combustion behavior was evaluated using a Vouch-6810 cone calorimeter according to ISO 5660, under a radiant heat flux of 50 kW/m^2^, using specimens with dimensions of 100 × 100 × 4 mm^3^. The cone calorimeter tests were carried out on three samples and the results averaged.

From the results of cone calorimeter tests, the following quantitative parameters were calculated: (i) SEA (specific smoke extinction area) indicates the total smoke produced per unit of mass lost and measures the smoke production efficiency;^16^ (ii) EHC (effective heat of combustion) is the heat released per kilogram of material burned and reflects the combustion efficiency of the pyrolysis gases;^16^ (iii) MARHE (maximum average rate of heat emission) represents the highest rate of heat released during a fire and provides insight into the intensity of the flame.^17^

The morphology of the samples was observed using scanning electron microscopy (SEM, Apreo C, Thermo Scientific). The obtained SEM micrographs were further analyzed using the software ImageJ.

Raman spectra were recorded at room temperature with a DXR2 Raman microscope (Thermo Scientific) employing a 532 nm excitation laser.

The mechanical properties were evaluated using an RGT-20A universal testing machine (Reger) with a crosshead speed of 25 mm/min for tensile tests and 15 mm/min for flexural tests. The impact strength was evaluated through notched Izod impact tests with a ZwickRoell HIT25P equipped with a 5.5 J pendulum. All the mechanical properties were carried out on five samples, and the results were averaged.

## 3 Results and Discussion

### 3.1 Microstructure Assessment of IFR-Containing Materials

As widely reported, the rheological behavior and the processability of a polymeric system are strongly affected by the thermomechanical degradation occurring during mechanical recycling processes^18^ and by the introduction of fillers.^19^ Here, dynamic rheological measurements were employed to evaluate the possible structural modification of the PP chains induced by recycling and to gain some information about the morphology of the IFR-containing materials. In Figure 1a, the complex viscosity curves as functions of the frequency are reported. Unfilled virgin PP (vPP) exhibits the typical Newtonian plateau at low frequencies, followed by a slight shear thinning and thus a decrease of the complex viscosity as the frequency increases. As a consequence of thermomechanical degradation that occurs during the reprocessing cycles, the complex viscosity of unfilled rPP decreases over the entire frequency range. Furthermore, an amplification of the Newtonian behavior can be clearly observed. Both of these features can be ascribed to the decrease in the PP molecular weight resulting from the chain-scission reactions occurring during the reprocessing steps.^18^

**Figure 1:**
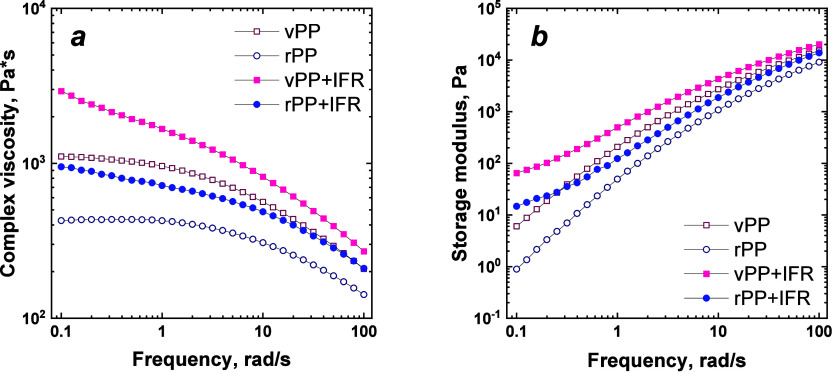
Complex viscosity (a) and storage modulus (b) for all investigated materials.

Concerning the rheological response of the materials containing IFRs, the addition of the additive results in an increase in the complex viscosity in the whole investigated frequency range. Furthermore, the Newtonian plateau almost disappeared for both systems, resulting in a sharp increase of viscosity at low frequencies and the appearance of yield stress behavior. The embedded solid particles, in fact, can restrict the mobility of the polymer chains, thereby hampering their complete relaxation. The effects of the IFR on the mobility of polymeric chains can also be recognized in the trend of the storage modulus as a function of the frequency, as depicted in Figure 1b. In fact, for both filled systems, the storage modulus increases in the low-frequency region, tending to become frequency-independent; once again, this behavior can be related to the alteration of the macromolecular dynamics resulting from the establishment of polymer/particles and particle/particle interactions.

The SEM micrographs of the IFR-containing materials are displayed in Figure 2a,b. From a general point of view, the IFR particles appear homogeneously distributed in both virgin and reprocessed polymers. In order to gain more information about the morphology of the two samples, the distribution of the particles’ diameter was evaluated considering the Feret diameter, which is extensively employed to measure particles with irregular shape and micrometric dimensions.^20^ Comparing Figure 2c with Figure 2d, reporting the distribution of the diameters in vPP- and rPP-based materials, respectively, different trends can be noticed. In particular, in rPP+IFR the distribution of particles’ diameter is shifted toward lower values and a lower average diameter is obtained, passing from 1.85 ± 0.07 μm to 1.34 ± 0.15 μm for vPP+IFR and rPP+IFR, respectively. This result, in agreement with the literature,^21,22^ can be ascribed to the lower viscosity of rPP as compared to the virgin matrix, which allows a finer and more homogeneous distribution of the embedded IFR particles.

**Figure 2:**
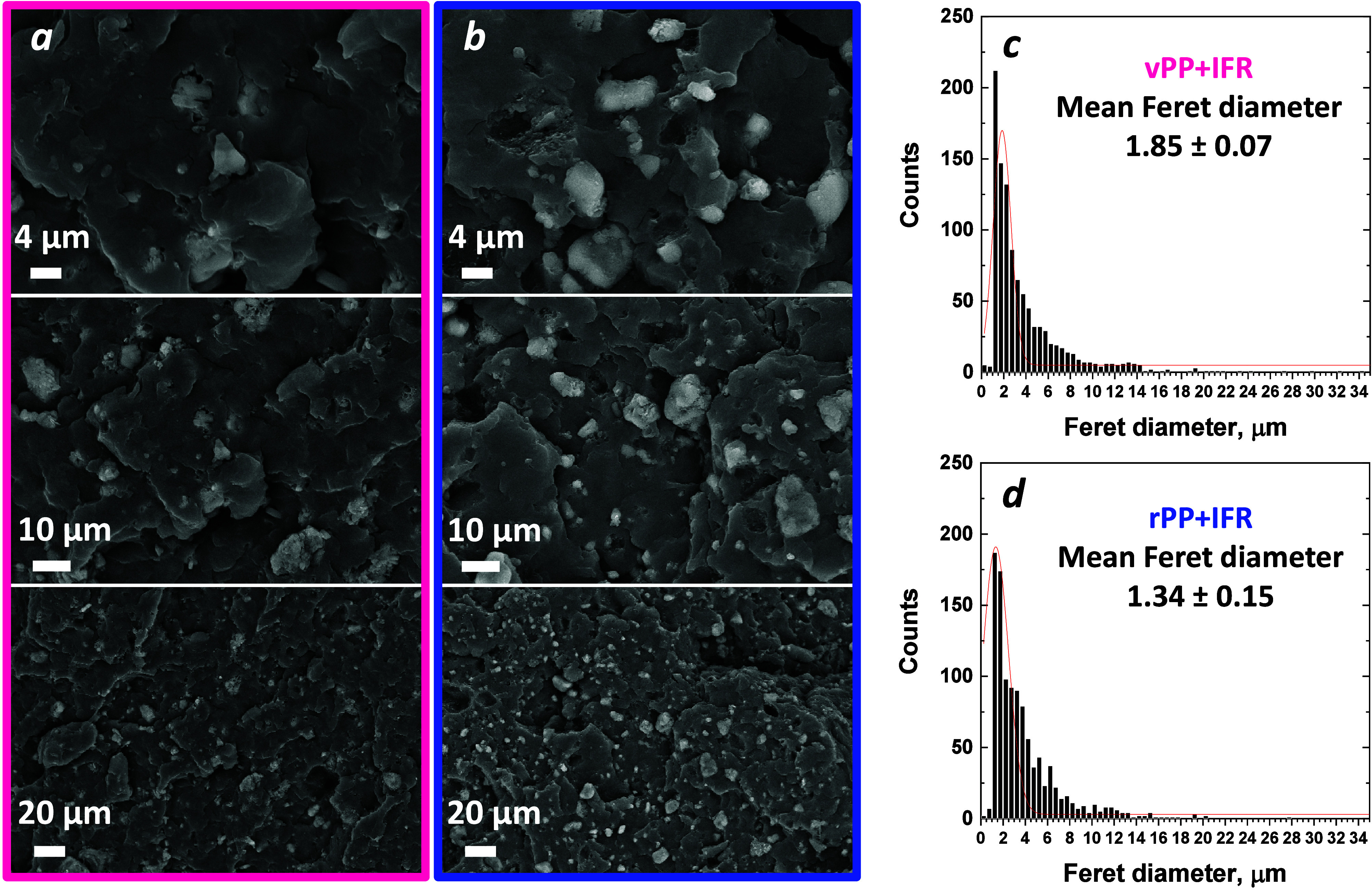
SEM micrographs of vPP+IFR (a) and rPP+IFR (b); distribution of IFR particles’ Feret diameter of vPP+IFR (c) and rPP+IFR (d).

### 3.2 Thermo-Oxidative Degradation of PP/IFR Composites

The thermo-oxidative degradation of unfilled and IFR-containing virgin and recycled PP was investigated through TGA tests in air atmosphere, and the obtained results are reported in Figure 3 and Table 1. As observable from Figure 3a,b, both unfilled virgin and recycled PP show one degradation step with no char residue at the end of the analysis. Additionally, despite recycled PP exhibits slightly early degradation onset as compared to the virgin polymer, the temperatures of maximum degradation of the two materials are practically coincident, indicating that the thermo-mechanical degradation occurred during the reprocessing cycles did not affect the PP thermo-oxidative stability. The IFR-containing materials, irrespective of the degradation level of the matrix, show the same main degradation step of unfilled PP, indicating that the introduction of IFR did not influence the main decomposition mechanism of PP, notwithstanding the occurrence of a second degradation step at higher temperatures, due to the IFR reactions.^10–13^ Furthermore, it should be noticed that the onset temperatures are practically unaffected by the introduction of IFR, while, as expected, *T*_max_ is slightly lower for the flame-retardant systems.

**Figure 3:**
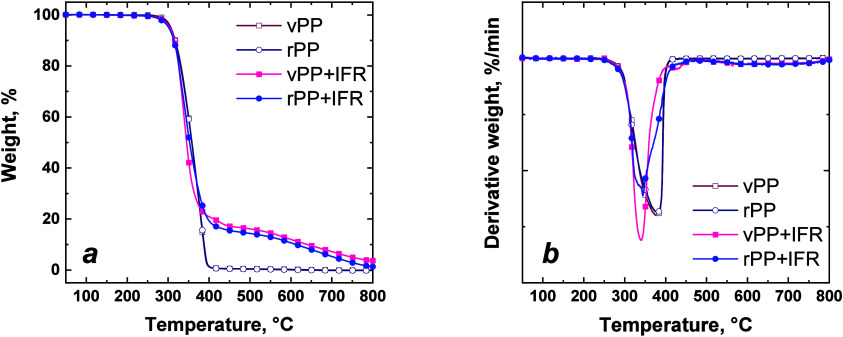
TGA (a) and dTG (b) curves of unfilled and IFR-containing virgin and recycled PP.

**Table 1:** Thermo-Oxidative Degradation Data for All Investigated Materials

	*T*_onset_ [°C]	*T*_max_ [°C]	residue at 600 °C [%]
vPP	294.5 ± 0.3	379.0 ± 0.1	0.20 ± 0.02
rPP	289.7 ± 0.2	381.2 ± 0.2	0.10 ± 0.03
vPP+IFR	287.4 ± 0.3	340.5 ± 0.2	12.0 ± 0.1
rPP+IFR	283.6 ± 0.4	344.1 ± 0.1	11.5 ± 0.1

### 3.3 Flame-Retardant Performances

#### 3.3.1 Flammability Evaluation

In order to estimate the flame-retardant performances of IFR-containing materials and their flammability, limiting oxygen index (LOI) and vertical burning (UL-94) tests were carried out. The obtained results for all of the investigated materials are reported in Table 2. For unfilled PP, whether virgin or recycled, no rating (NR) was achieved during vertical burning tests; in addition, similar LOI values were achieved for both samples. As expected, both the IFR-containing materials achieved a V-0 classification, and increased values of LOI are observed as compared to unfilled PPs. During vertical burning tests, both materials stopped burning after the first ignition, and during the second application of the flame, they burned for a limited time without any dripping. No significant differences were observed for FR vPP or rPP, suggesting that the used IFR system was effective in improving the fire performance of recycled PP as well.

**Table 2:** UL-94 Vertical Burning and LOI Results

	rating	*t*_1_ [s]	*t*_2_ [s]	dripping	LOI [%]
vPP	NR	5.4 ± 2.7	>30	yes	21
rPP	NR	4.6 ± 2.1	>30	yes	20.5
vPP+IFR	V-0	0	3.5 ± 1.0	no	32.5
rPP+IFR	V-0	0	3.9 ± 1.5	no	33.4

#### 3.3.2 Combustion Behavior

The results obtained from cone calorimeter tests are reported in Table 3 and Figure 4. As observable in Figure 4a from the curves of heat release rate (HRR), both virgin and recycled PPs exhibit the same combustion behavior. In particular, both samples are characterized by similar time to ignition (TTI), after which the HRR curves show a sharp increase followed by a fast decrease with the flame being out in about 190 s. Furthermore, during the combustion, a complete consumption of the entire sample occurs, with a decrease of the specimen mass almost immediate (Figure 4d).

**Figure 4:**
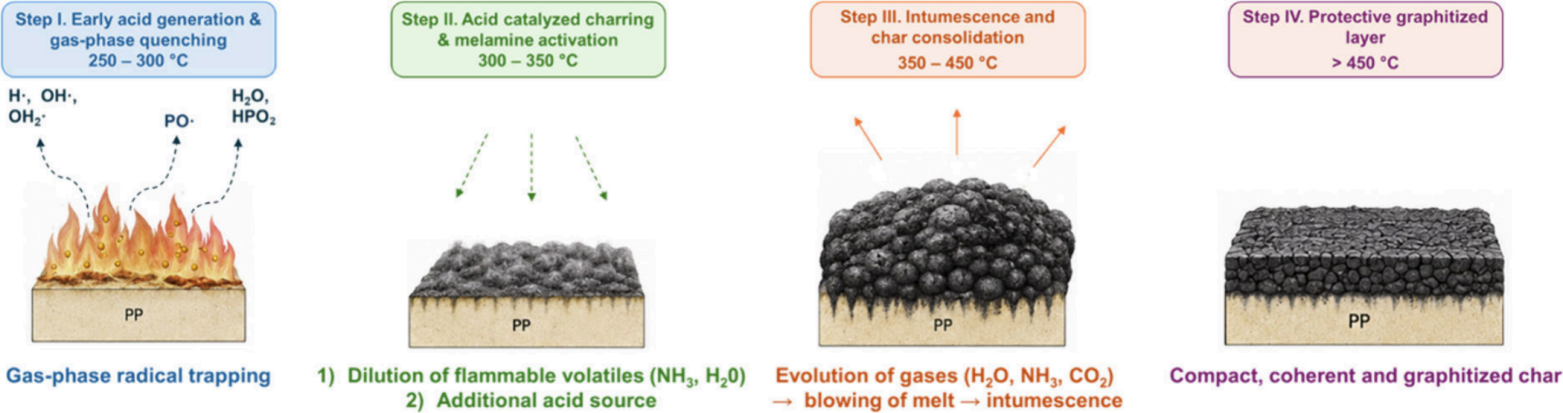
Condensed- and gas-phase acting mechanism of the IFR in PP.

**Table 3:** Cone Calorimeter Tests Results of Unfilled and FR Virgin and Recycled PP

	vPP	rPP	vPP+IFR	rPP+IFR
TTI [s]	34 ± 5	32 ± 3	13 ± 2	20 ± 1
ignition to flameout [s]	189 ± 10	187 ± 23	933 ± 153	1738 ± 60
peak_1_-HRR [kW/m^2^]	3406 ± 194	3445 ± 151	338 ± 35	349 ± 45
t peak_1_-HRR [s]	178 ± 9	180 ± 2	308 ± 8	246 ± 22
peak_2_-HRR [kW/m^2^]			451 ± 17	279 ± 46
t peak_2_-HRR [s]			819 ± 126	1514 ± 161
THR [MJ/m^2^]	263 ± 6	265 ± 1	200 ± 2	183 ± 10
peak_1_-SPR [m^2^/s]	0.191 ± 0.011	0.198 ± 0.028	0.023 ± 0.005	0.025 ± 0.007
t peak_1_-SPR [s]	167 ± 6	182 ± 2	297 ± 25	232 ± 15
peak_2_-SPR [m^2^/s]			0.067 ± 0.013	0.045 ± 0.013
t peak_2_-SPR [s]			801 ± 137	1495 ± 223
TSP [m^2^]	17.1 ± 0.5	18.5 ± 1.6	19.2 ± 2.8	17.7 ± 2.8
TSR [m^2^/m^2^]	2108 ± 58	2276 ± 208	2352 ± 334	2188 ± 351
char residue [wt %]	1.2 ± 0.5	1.6 ± 0.4	11.3 ± 0.4	10.8 ± 0.8
SEA [m^2^/kg]	651 ± 8	758 ± 42	604 ± 27	433 ± 53
EHC [MJ/kg]	55.1 ± 3.2	55.9 ± 1.8	48.1 ± 3.2	34.1 ± 3.0
MARHE [kW/m^2^]	1264 ± 73	1255 ± 30	220 ± 32	155 ± 20
MLR [g/s]	0.140 ± 0.007	0.139 ± 0.017	0.030 ± 0.005	0.015 ± 0.001

Looking at the results of the cone calorimeter tests reported in Table 3, no substantial differences are observed, suggesting that the thermomechanical degradation of PP, and the consequent microstructural modifications, did not affect its combustion behavior.

Due to the incorporation of IFR, for both vPP- and rPP-based samples, the heat release significantly decreases. Additionally, the HRR curves display two main peaks, attributable to the typical behavior of char-forming material.^15,23,24^ In particular, the first peak can be attributed to ignition and flame spread on the surface. After this, a plateau effect occurs because of the formation of a protective char layer. The second peak, which appears at a longer time, is associated with the rupture of the char layer caused by volatile gases escaping from the degradation of the substrate.

Specifically, the flame-retardant action of PAPP and MPP, as already described in the literature,^11^ proceeds through four stages, acting in both the gas and condensed phases (as also depicted in Figure 4). The first step (occurring at about 250–300 °C) concerns the early acid generation and gas-phase quenching. In particular, the partial depolymerization of PAPP liberates poly-/pyrophosphoric acids together with PO^•^ radicals. The acids initiate dehydration of oxidized PP chain ends, while the phosphorus-centered radicals scavenge H^•^ and OH^•^ in the flame, lowering the effective heat of combustion. As the temperature increases, reaching about 300–350 °C, the newly formed acids react with residual P–OH and −NH^+^_2_–OP moieties in PAPP and with MPP, producing melamine poly-phosphate salts and generating branched P–O–P and P–N–C frameworks. Simultaneously, NH_3_ and H_2_O are released, diluting the combustible volatiles. The further step, which takes place at about 350–400 °C, involves intumescence and char consolidation. In fact, the continued cross-linking of the P–N–C/P–O–P network and the foaming action of the inert gases give rise to an expanded yet cohesive intumescent layer. Finally, above about 450 °C, progressive aromatization and condensation of this phosphorus-rich matrix convert the foamed layer into a dense, highly graphitised char that efficiently blocks heat and mass transfer.

As clearly observable from the results reported in Table 3 and Figure 5a, vPP+IFR exhibits an 87% reduction of the peak of HRR (pkHRR) as compared to the unfilled matrix, with an expected decrease of the TTI, due to the accelerated decomposition of the polymeric matrix promoted by IFR. However, a dramatic increase of the time from ignition to flameout as compared to vPP can be clearly observed. Furthermore, the incorporation of IFR in vPP induced a reduction of about 24% (roughly corresponding to the amount of embedded IFR) of THR (Figure 4b), and the mass loss rate (Figure 5d) for this sample was significantly reduced compared to the unfilled matrix.

**Figure 5:**
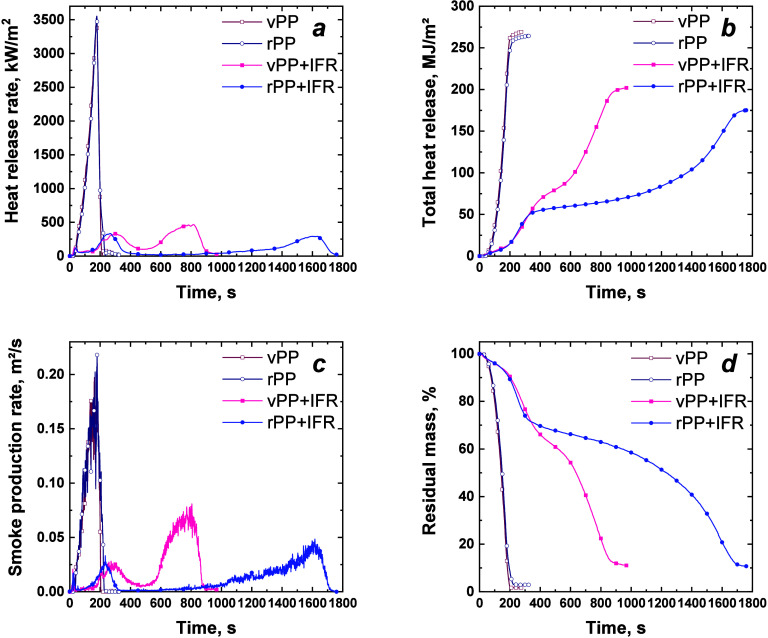
Cone calorimeter results: heat release rate (a), total heat release (b), smoke production rate (c), and residual mass (d).

Notably, the incorporation of IFR into rPP induces a markedly different combustion behavior. Specifically, the HRR of the rPP+IFR formulation decreases by approximately 90% compared to that of neat rPP. Although ignition occurs earlier than in the unfilled polymer, the TTI remains higher than that observed for the vPP+IFR sample. More importantly, while the first HRR peak appears at nearly the same time as in vPP+IFR, the onset of the second peak is substantially delayed in rPP+IFR (Figure 5a). This result indicates that the char layer formed after ignition in the rPP-based system remains stable for up to approximately 800 s, effectively suppressing flame propagation. Additional evidence of the enhanced stability of the char formed in this sample is provided by the much slower increase in THR (Figure 5b) and the reduced mass loss rate compared to the vPP-based counterpart. In addition, regarding THR, it should be noted that a reduction of approximately 31% was achieved for rPP+IFR in comparison with neat rPP. This result indicates that in this case approximately 10% of the polymer underwent charring during the tests, thereby preventing it from contributing to the combustion process.

Besides, as observable from the results reported in Table 3, lower values of SEA, EHC, and MARHE were obtained for rPP+IFR. Thus, when introducing the PAPP:MPP mixture in recycled PP, a lower amount of smoke was generated (lower SEA), lower energy was released from the material when it burned owing to an improved flame inhibition (lower EHC), and the fire safety was enhanced since the fire growth potential is reduced (lower MARHE).

All the previous considerations ensure the effectiveness of this kind of intumescent flame-retardant into recycled PP, and even more importantly, it appears that the used IFR system is able to provide enhanced effectiveness when employed in recycled PP as compared to its use in virgin polymer. This surprising result can be explained considering two different phenomena. On one hand, the initial melt viscosity of the material can influence the evolution of melt rheology during fire exposure. In fact, melt viscosity is known to strongly affect polymer combustion behavior and flame retardancy.^25^ The recycled PP-based system exhibits lower viscosity and shorter polymer chains if compared to its virgin counterpart due to chain scission reactions occurring during the reprocessing. Therefore, a lower initial viscosity, typically associated with reduced molecular weight and increased chain mobility, may facilitate radical recombination reactions during thermal degradation, promoting a better reactivity with the IFR system. This could promote condensed-phase stabilization and char formation, contributing to improved flame retardancy.^1^ On the other hand, the results could be also explained considering the different extent of distribution of the IFR particles in virgin or recycled PP. More specifically, as assessed from the morphological characterization, IFR is better dispersed in rPP, owing to its lower viscosity as compared to vPP, thus leading to improved fire performances. In fact, according with literature,^22,26–29^ a more uniform dispersion of the flame retardant additive, resulting in particles of smaller dimensions embedded in the polymeric matrix, promotes a faster formation of a continuous intumescent charred layer, thereby resulting in enhanced fire performances.

In order to assess the fire safety performances, thus the fire intensity and the fire hazard, two parameters are usually evaluated, namely, fire performance index (FPI = TTI/pkHRR) and fire growth rate index (FIGRA = pkHRR/tpkHRR). The lower the intensity of the fire, the higher is the FPI value; on the other hand, for a lower fire hazard, a lower value of FIGRA is required. Therefore, for polymer-based systems, a high FPI and a low FIGRA usually imply great fire safety.^10^ These indexes have been calculated for both unfilled and FR/PPs, and the obtained values are reported in Table 4. As expected, neat virgin and recycled PP showed very low values of FPI and very high values of FIGRA. With the introduction of IFR, both vPP+IFR and rPP+IFR exhibited an increase in the FPI and a decrease in FIGRA, revealing the achievement of increased fire safety performance as compared to the unfilled matrices.

**Table 4:** FPI, FIGRA, and FRI Values

	FPI [m^2^·s/kW]	FIGRA [kW/m^2^/s]	FRI
vPP	0.010 ± 0.003	19.2 ± 2.0	
vPP+IFR	0.040 ± 0.005	1.10 ± 0.13	5.1
rPP	0.009 ± 0.001	19.2 ± 1.1	
rPP+IFR	0.058 ± 0.009	1.43 ± 0.28	8.9

The flame retardancy index (FRI) is also reported in Table 4. FRI is a dimensionless index that quantifies the flame-retardancy ability of polymeric systems, taking the neat polymer as a reference sample.^30^ An FRI value between 10^0^ and 10^1^ represents good flame-retardancy performance, and as shown in Table 4, the FRI values of both IFR-containing materials lie in that range. However, the FRI of rPP+IFR was higher than that of vPP+IFR, suggesting, once again, better flame-retardancy performance for the rPP-based system.

#### 3.3.3 Char Analysis

The char layer collected at the end of the cone calorimeter tests was also characterized. It should be pointed out that, due to the different times of flame-out of the IFR-containing materials, the condensed-phase analyses have been carried out after about 900 s in the case of vPP+IFR and after about 1700 s for rPP+IFR. The evolution of the char during the tests was comparable for the two samples; however, the longer time elapsed from the ignition to the flameout for recycled PP-based materials affected the final morphology of the char residues.

The pictures of the residual char of virgin and recycled PP-based materials are shown in Figure 6. It can be clearly observed that vPP+IFR formed a relatively high intumescent char layer with few holes on the outer surface. On the other hand, the height of the residue collected for rPP+IFR was about half that of its virgin PP-based counterpart. In both samples, the presence of holes could be ascribed to the decomposition reaction of MPP that, producing inert gases, causes the breakage of the char layer.^31^ The differences in the height of the char residues can be attributed to the remarkably different time intervals in which the combustion takes place for the two samples (about 13 min longer for rPP+IFR). However, as demonstrated in the literature,^5^ the noticed difference in the char height should not affect the fire performances. The evaluation of the char morphology through SEM characterization (see micrographs reported in Figure 6e,f) highlights that for vPP-IFR the outer layer has a dense structure with few holes, while the inner layer is characterized by a porous structure with some microsized channels. On the other hand, these differences between the outer and the inner surfaces were not observed for rPP+IFR. In particular, in this last case, a less dense structure for the outer layer and a more closed and compact structure for the inner layer can be recognized.

**Figure 6:**
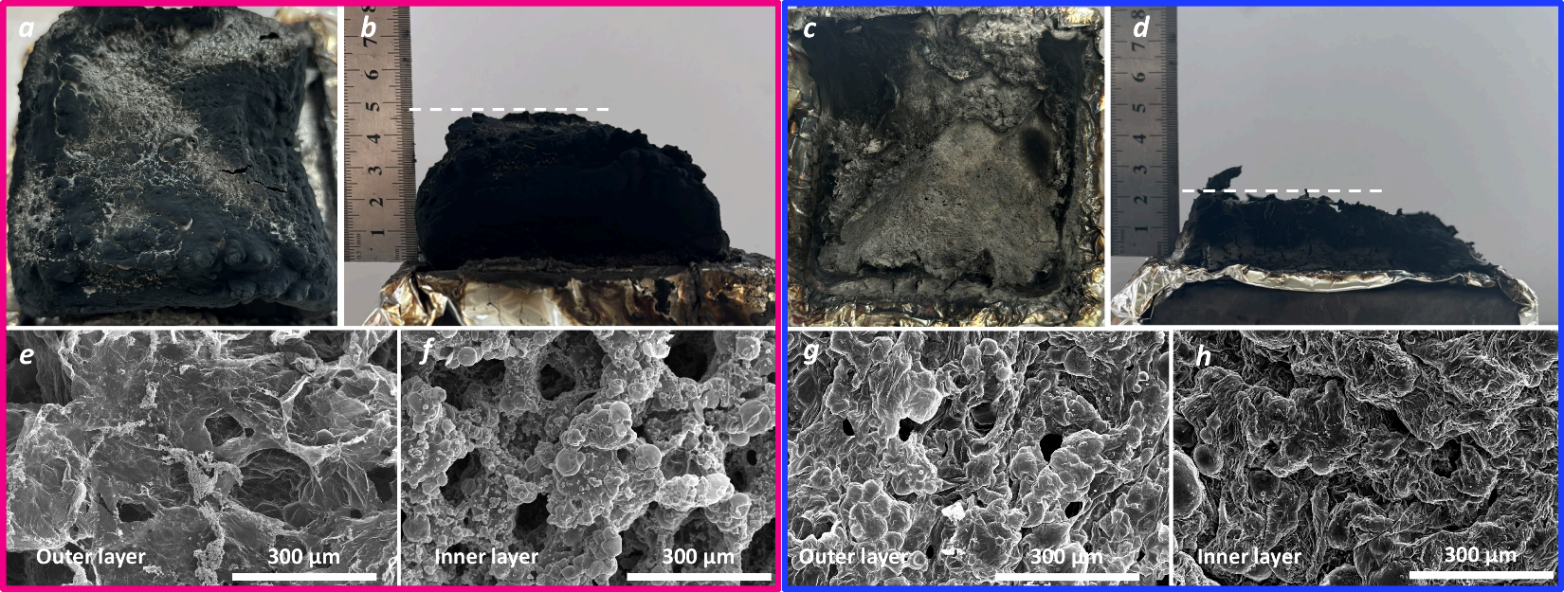
Pictures and SEM images of residual char: (a, b, e, f) vPP+IFR; (c, d, g, h) rPP+IFR.

The more compact structure of the char in rPP+IFR can be invoked to explain the reduced fire growth during the cone calorimeter test noticed for this sample. In fact, the compact char can hinder the diffusion of heat and volatiles in a more efficient way as compared to the porous structure of vPP+IFR, which instead provides a more rapid mass (oxygen and pyrolysis products) and heat transport.

The observed different morphology of the char can be correlated with the distribution and the dimension of the IFR particles; in fact, as already mentioned by other authors, a more compact and dense structure of char usually results from materials containing low-viscosity polymers and better-distributed smaller FR particles.^22,27^

Finally, the graphitized structure of the residual char was analyzed by Raman spectroscopy. In particular, the degree of graphitization was quantitatively determined by calculating the ratio between the peak areas of the defect and graphite bands (*I*_D_/*I*_G_), located in the range 1350–1380 cm^–1^ and 1580–1600 cm^–1^, respectively. The fitted Raman spectra of both systems are reported in Figure S1. In general, the *I*_D_/*I*_G_ ratio is widely used to describe the structural organization of carbonaceous materials, where lower values indicate a higher degree of graphitization (i.e., more ordered carbon structures), while higher values are associated with increased structural disorder.^32,33^ Moreover, a high degree of graphitization of the residues (i.e., lower values of the ratio I_D_/I_G_) is associated with better fire performances.^32–34^ A lower *I*_D_/*I*_G_ (namely, 0.7) was obtained for rPP+IFR when compared to vPP+IFR (*I*_D_/*I*_G_ = 1.2), indicating that the char layer formed in the recycled PP-based system is more graphitized and structurally ordered. This higher degree of graphitization results in a denser and more cohesive char, which is more effective in limiting heat transfer and suppressing the release of volatile degradation products. These findings are consistent with the fire performance observed in cone calorimeter tests and with the morphological characterization of the char residues. In particular, SEM analysis qualitatively confirms the formation of a more compact and continuous protective layer in rPP+IFR. Therefore, the results indicate that the intumescent carbon layer of rPP+IFR exhibits enhanced structural integrity and barrier functionality, effectively reducing heat transmission from the combustion zone to the underlying polymer matrix and contributing to improved flame-retardant performance.

### 3.4 Mechanical Properties

Lastly, in order to assess the actual structural usability of flame-retardant materials, the mechanical behavior of the samples was characterized through bending, tensile, and impact tests. In fact, it is well-known that the introduction of FR additives usually induces a worsening of the mechanical properties of the material, particularly affecting its deformability.^22,35–38^ Additionally, this issue is even more important for recycled polymers, considering that the degradation underwent from the material during the reprocessing usually results in a severe worsening of the mechanical performance as compared to its virgin counterpart.^38^

As reported in Figure 7 and Table 5, vPP+IFR and rPP+IFR show practically comparable values of tensile strength and flexural strength. Conversely, the impact strength and the elongation at break decrease when rPP is used as matrix, likely due to the typical embrittlement shown by recycled polymers.^39^ Nonetheless, the results indicate that despite the presence of the IFR and the use of a recycled polymer, the rPP+IFR system exhibits adequate mechanical properties for a broad spectrum of potential applications.

**Figure 7:**
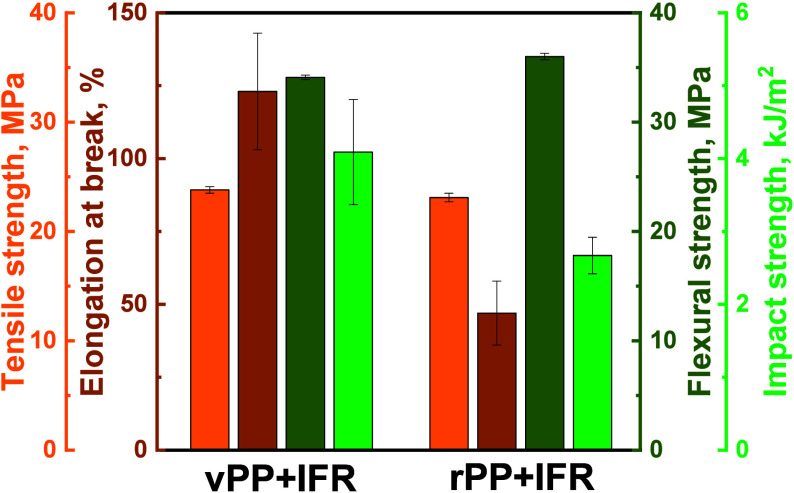
Mechanical properties of vPP- and rPP-based systems.

**Table 5:** Specific Values of Mechanical Properties of vPP- and rPP-Based Systems

	tensile strength [MPa]	flexural strength [MPa]	impact strength [kJ/m^2^]	elongation at break [%]
vPP+IFR	23.8 ± 0.3	34.1 ± 0.2	4.1 ± 0.7	123 ± 20
rPP+IFR	23.1 ± 0.4	36.0 ± 0.3	2.7 ± 0.3	47 ± 11

## 4 Conclusions

In this work, the effectiveness of an intumescent flame-retardant system for recycled PP-based materials was evaluated and compared with that of its virgin PP-based counterpart. Both materials were melt-compounded with 21 wt % of a PAPP/MPP mixture (2:1) and characterized for rheological, morphological, mechanical, and fire-performance properties. Owing to the lower viscosity of the recycled polymer, the rPP+IFR sample showed more finely and uniformly dispersed IFR particles, which contributed to the improved combustion behavior observed in cone calorimeter tests. Despite the comparable decrease in the heat release rate observed for vPP+IFR and rPP+IFR, the system based on rPP demonstrated a higher time to ignition and especially a more stable char layer, which resulted in a substantial delay in the occurrence of the second HRR peak. Lower SEA, EHC, and MARHE values further indicated enhanced fire safety for rPP+IFR. Furthermore, both systems achieved a V-0 rating in UL-94 and comparable LOI values. Additionally, the evaluation of the mechanical behavior of the samples demonstrated that the utilization of rPP as a matrix did not compromise the tensile and the flexural strength of the material, despite a slight decrease in ductility and impact strength. Overall, this study demonstrates that recycled PP can effectively support intumescent flame-retardant systems, thereby enabling high-performance flame-retardant materials while promoting circular-economy strategies and reducing reliance on virgin PP. Furthermore, the findings provide a fundamental basis for understanding the performance of these systems in reprocessed polypropylene under controlled conditions, thus paving the way for future investigations on more industrially representative recycled PP streams, as well as on processing stability and large-scale applicability.

## Supplementary Material



## Notes

Figure S1 showing fitted Raman spectra of residual char of vPP+IFR and rPP+IFR (PDF)
