# Supplementary material for: Is Recycled Polypropylene Suitable for Flame-Retarded Applications?
Source: ACS Appl Polym Mater. 2026 Jun 12;8(12):9332–41. doi: 10.1021/acsapm.6c00885 (PMC13317669; doi:10.1021/acsapm.6c00885)
Supplement: Supplementary file 1 [file ap6c00885_si_001.pdf]

SUPPORTING INFORMATION

Is Recycled Polypropylene Suitable for Flame-Retarded Applications?

Giulia Bernagozzi[1,2], Rossella Arrigo[1,2], Yue Xu[3], Miaojun Xu[3], Alberto Frache[1,2]*

[1] Department of Applied Science and Technology, Politecnico di Torino, Viale Teresa Michel 5, 15121, Alessandria, Italy

[2] National Interuniversity Consortium of Materials Science and Technology (INSTM), Via G. Giusti 9, 50121, Firenze, Italy

[3] Heilongjiang Key Laboratory of Molecular Design and Preparation of Flame Retarded Materials, College of Chemistry, Chemical Engineering and Resource Utilization, Northeast Forestry University, No. 26, Hexing Road, Xiangfang District, 150040, Harbin, China

* Corresponding author: alberto.frache@polito.it

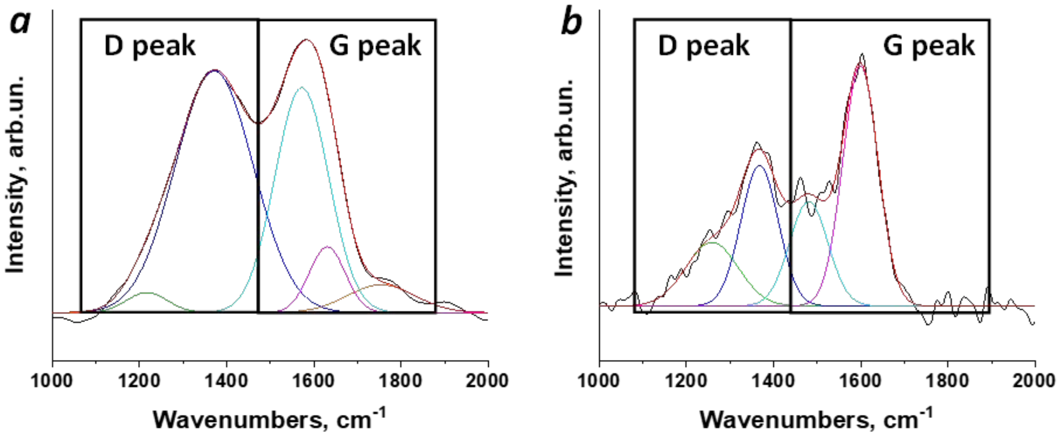

Figure S1. Fitted Raman spectra of residual char of vPP+IFR (a) and rPP+IFR (b).
